# The Omission of Accent Marks Does Not Hinder Word Recognition: Evidence From Spanish

**DOI:** 10.3389/fpsyg.2021.794923

**Published:** 2021-12-13

**Authors:** Ana Marcet, María Fernández-López, Melanie Labusch, Manuel Perea

**Affiliations:** ^1^Department of Language and Literature Teaching, Universitat de València, Valencia, Spain; ^2^Department of Methodology of Behavioral Sciences and ERI-Lectura, Universitat de València, Valencia, Spain; ^3^Center of Research in Cognition, Universidad Antonio de Nebrija, Madrid, Spain

**Keywords:** word recognition, lexical access, reading, lexical decision, accent marks

## Abstract

Recent research has found that the omission of accent marks in Spanish does not produce slower word identification times in go/no-go lexical decision and semantic categorization tasks [e.g., cárcel (prison) = carcel], thus suggesting that vowels like á and a are represented by the same orthographic units during word recognition and reading. However, there is a discrepant finding with the yes/no lexical decision task, where the words with the omitted accent mark produced longer response times than the words with the accent mark. In Experiment 1, we examined this discrepant finding by running a yes/no lexical decision experiment comparing the effects for words and non-words. Results showed slower response times for the words with omitted accent mark than for those with the accent mark present (e.g., cárcel < carcel). Critically, we found the opposite pattern for non-words: response times were longer for the non-words with accent marks (e.g., cárdil > cardil), thus suggesting a bias toward a “word” response for accented items in the yes/no lexical decision task. To test this interpretation, Experiment 2 used the same stimuli with a blocked design (i.e., accent mark present vs. omitted in all items) and a go/no-go lexical decision task (i.e., respond only to “words”). Results showed similar response times to words regardless of whether the accent mark was omitted (e.g., cárcel = carcel). This pattern strongly suggests that the longer response times to words with an omitted accent mark in yes/no lexical decision experiments are a task-dependent effect rather than a genuine reading cost.

## Introduction

One of the most characteristic features of written Spanish—together with the letter ñ—is the presence of acute accents in words. These accent marks indicate, under some rules, which one is the stressed vowel in the word [e.g., mítico (mythic); lápiz (pencil); camión (truck); see Marcet and Perea, 2021, for an overview of the rules of accentuation in Spanish; see also Real Academia Española, 2010, for a more detailed description].

Whether or not accent marks—also called diacritics—help silent reading in Spanish has been highly debated in the past decades. Indeed, many renowned writers and scholars have advocated for a much more lenient use of accent marks in a language where more than 80% of words have their stress in the last-but-one syllable (Quilis, 1993). The best example is probably the speech given by Gabriel García-Márquez at the International Conference of Spanish Language in Zacatecas in 1997. Indeed, other Romance languages such as Italian or Romanian have a much sparer role for accent marks. For instance, accent marks in Italian are mostly used for polysyllabic words with a stressed final vowel [e.g., libertà (freedom)] or to tell apart otherwise homonym words with a different accented syllable [e.g., àncora (anchor) vs. ancora (still)] (see Colombo and Sulpizio, 2021).

Besides the debates on the practical function of accent marks, the existence of accent marks in a given language raises a fundamental theoretical question: Should accented and non-accented vowels be treated as different orthographic representations? Computational models assume an English orthography in which the vowels á and a would be treated as a single orthographic unit (e.g., multiple read-out model: Grainger and Jacobs, 1996; spatial coding model: Davis, 2010). For instance, to simulate data from Spanish in the multiple read-out model, Conrad et al. (2010) removed the accent marks in diacritical words [e.g., the word ratón (mouse) was encoded as raton]. There is, however, a computational model, devised for French (Ans et al., 1998, multiple-trace model), where each diacritical vowel is represented differently (e.g., é, è, and e would be considered separate orthographic representations).

We believe that the answer to the above question is probably language-dependent (see Wells, 2000; Chetail and Boursain, 2019; Marcet et al., 2021): accent marks are probably represented as different abstract representations in languages where they indicate vowel quality (e.g., Finnish: Perea et al., 2021a; German: Perea et al., 2021b), but not in languages where accent marks only indicate lexical stress with no change in vowel quality (Spanish: Perea et al., 2020b). Concerning Spanish, which is the focus of the present study, recent empirical evidence with adult readers has shown that accent marks do not help the initial encoding of words. In a masked priming lexical decision experiment, Perea et al. (2020b) found that the response times to a word like FÁCIL (easy) were essentially the same when it was preceded by the identity prime fácil or the prime facil (i.e., with an omitted accent). In contrast, the control prime fécil was the least effective. If á and a activate different orthographic representations, one would have expected faster responses in the identity priming condition over the other two priming conditions. Likewise, in a silent sentence reading task where the participants’ eye movements were recorded, Marcet and Perea (2021) found that first-pass measures on a target word [first-fixation duration, gaze duration (sum of first-pass fixations including refixations)] were remarkably similar regardless of whether the accent mark was present [e.g., cárcel (prison) or omitted (carcel)]. Furthermore, Perea et al. (2021b) found a similar pattern using a semantic categorization task (“was the word an animal name or not?”): word recognition times were extremely similar regardless of whether the accent mark was present or not [e.g., ratón (mouse) = raton; cárcel = carcel].

Notably, the empirical evidence using a single-presentation lexical decision task (i.e., a word/non-word discrimination task) in Spanish is contradictory. In a yes/no lexical decision task, Schwab (2015, Experiment 1) found faster responses to the words with the accent present than those with the accent omitted (29 ms: 761 vs. 790 ms, respectively). Although the difference was not significant (the *t*-value in the linear mixed-effects model was 1.55), this was probably due to the experiment being underpowered—the number of observations per condition was only 220 (22 participants and ten items/condition). Another interpretive issue was that the data for the non-words (e.g., the comparison of the non-words lámiz vs. lamiz) was not reported or analyzed. To reach firm conclusions, one would need to examine both word and non-word data. The logic is that, in this scenario, items without accent marks (e.g., carcel and lamiz) could have been less “wordlike” than the items with accent marks (e.g., cárcel and lámiz). If so, the omission of accent marks would produce slower response times to words but faster response times to non-words (see Perea et al., 2020a, for evidence of biases due to stimulus format in the lexical decision task). In a second experiment, Schwab (2015) employed a go/no-go lexical decision task (i.e., participants responded to “words” but not to “non-words”) where the “accent present” items (e.g., words like cárcel and non-words like lámiz) and “accent omitted” items (e.g., carcel and lamiz) were shown in separate blocks. In this scenario, word response times were remarkably similar for cárcel and carcel. Schwab concluded that accent marks might not be necessary for Spanish words, at least for those with unambiguous spelling (i.e., words that do not create other words when removing the accent mark). However, there was no attempt to solve the differences between Experiments 1 and 2.

The goal of this paper was to resolve the apparent discrepancies regarding the reading cost due to the omission of accent marks in Spanish in the yes/no lexical decision task. To obtain the full picture, we examined the word and non-word data in a yes/no lexical decision task (Experiment 1) and in a go/no-go lexical decision task (Experiment 2). The rationale is that the advantage of cárcel over carcel reported by Schwab (2015, Experiment 1) in a yes/no lexical decision task could have been due to the accented items producing a “word” bias rather than a genuine task-independent advantage in word recognition. Of note, this mechanism would not be operative in Schwab (2015, Experiment 2) with the go/no-go procedure because of the blocked design (i.e., items with accent marks in one block vs. items without accent marks in the other block). We used the set of words from Marcet and Perea (2021) and Perea et al. (2021a) experiments for comparison purposes. Of note, these stimuli, which had an unambiguous spelling (i.e., the omission of the accent mark did not produce another word), only showed a negligible disadvantage of the words with the omitted accent marks.

Regarding the predictions of Experiment 1 (yes/no lexical decision task), we can envision three possible scenarios. The first scenario is that the findings with word stimuli in Schwab (2015, Experiment 1) were an empirical anomaly. If so, one would expect similar response times to cárcel and carcel. The second scenario is that, while the findings with word stimuli from Schwab (2015, Experiment 1) were reliable (i.e., faster responses to cárcel than to carcel), this apparent reading cost was due to a “word” bias for accented items in lexical decision (i.e., a task-specific effect). In this case, we would expect faster response times for the words with diacritics (cárcel faster than carcel) but slower response times for the non-words with diacritics (cárdil slower than cardil) (i.e., a “word” bias for accented items). The third scenario is to replicate the pattern of Schwab (2015, Experiment 1) word data, but with the non-words not showing an effect due to lack of diacritics (or faster responses for the non-words with diacritics). This last outcome would require rethinking the idea that the omission of diacritics in Spanish has little cost in lexical access.

## Experiment 1

### Materials and Methods

#### Participants

We recruited 40 university students, all native speakers of Castillian Spanish with normal/corrected vision and no reading problems, from the Prolific platform. This sample size guarantees 2,400 observations in each condition (40 participants × 60 items/condition), thus having enough power to detect small-sized effects (see Brysbaert and Stevens, 2018). In this and the following experiment, all participants signed an informed consent form at the beginning of the session, and the Ethics Committee of the Universitat de València approved the experiments.

#### Procedure

The experiment was conducted online using the Pavlovia server.^1^ The program was written with PsychoPy 3 (Peirce and MacAskill, 2018). LimeSurvey^2^ was also used to obtain demographic data before the experiment. Participants were instructed to do the experiment in a silent place without any interruptions. They received the usual lexical decision instructions: they had to decide whether the item on the screen was a Spanish word (if so, press M on the keyboard) or not (if so, press Z on the keyboard) as quickly and accurately as possible. A given trial started with a fixation cross that was presented for 500 ms in the center of the computer screen. Then, the item appeared in the same location as the fixation cross until the participant responded or until a deadline of 2 s. The order of trials was randomized for each participant. There was a short practice phase of sixteen trials before the experimental phase (240 trials). In the experimental phase, there were short breaks every sixty trials. The duration of the experiment was approximately 12–15 min.

#### Materials

The set of words was composed of the 120 accented Spanish words used by Marcet and Perea (2021). These words had a length between 5 and 10 letters (*M* = 6.4) and a Zipf frequency between 1.85 and 5.59 (*M* = 3.73) (Duchon et al., 2013). The position of the accent mark varied across words (last syllable, second-to-last syllable, third-to-last syllable; see Marcet and Perea, 2021, for further details). To create the set of 120 orthographically legal non-words of the same length as the word stimuli, we employed Wuggy (Keuleers and Brysbaert, 2010). As the output from Wuggy does not contain accent marks, we added them as in their base words [e.g., cáciro (baseword: cámara), cráror (baseword: crater), and sanión (baseword: ración)]. We created two lists of counterbalanced stimuli, each with half of the items being accented (e.g., if cráter were presented in List 1, crater would be presented in List 2). The list of stimuli (both words and non-words) is available in the same OSF link as the data (see “Data Availability” section).

#### Results and Discussion

In the analyses of the response times, we removed those latencies shorter than 250 ms and the incorrect responses. The mean correct response times and error rates (in percentage) per condition are presented in Table 1.

**TABLE 1:** Mean lexical decision times (in ms) and error rates (in percentages) for words and non-words with vs. without accent marks in Experiment 1.

	With accents		Without accents	
	Response time	% Errors	Response time	% Errors
**Words**	641	4.2	665	6.2
**Non-words**	736	4.4	710	3.6

For the inferential analyses, the latency and accuracy data were fitted with Bayesian linear mixed-effects models using the brms package (Bürkner, 2016) in the R environment (R Core Team, 2021). The fixed factors in the model were Format [without diacritics (−0.5), with accent mark (0.5)] and Lexicality [word (−0.5), non-word (0.5)]. Following Barr et al. (2013), we chose the maximal random-effect structure justified by the experimental design:


RT[accuracy]=FormatLexicality*+(1+FormatLexicality*|subject)+(1+Format|item).

The latency data were fitted with the exGaussian function to capture the positive skew of the response times, and the accuracy data were fitted with the Bernoulli function due to the inherent binary responses in each trial (correct = 1; error = 0). Each model received 5,000 iterations (1,000 as a warm-up). The models converged successfully and $\hat{R}$ = 1.00 in all parameters. The output of the Bayesian models does not provide a *p*-value for each effect; instead, they provide a 95% Credible Interval (95% CrI), together with an estimate of the parameter and its standard error, that can be interpreted as evidence of an effect when the interval does not cross zero.

In the latency model, we found evidence of main effects of Format and Lexicality [Format: *b* = 22.88, SE = 2.73, 95%CrI (17.51, 28.22), Lexicality: *b* = 69.28, SE = 8.61, 95%CrI (52.30, 86.11)]. More importantly, we also found evidence of an interaction between the two factors [*b* = −37.48, SE = 4.07, 95%CrI (−45.46, −29.45)]. This interaction reflected: (1) faster responses for the words with the accent present over the words with the accent omitted [95%CrI (−28.07, −17.4)], and (2) slower responses for the non-words with an accent present than for non-words with an accent omitted [95%CrI (8.61, 20.6)].

In the accuracy model, we did not find evidence of the effects of Format and Lexicality [Format: *b* = −0.34, SE = 0.19, 95%CrI (−0.70, 0.04), Lexicality: *b* = 0.11, SE = 0.27, 95%CrI (−0.41, 0.66)]. Notably, we found evidence of an interaction between the two factors [*b* = 0.69, SE = 0.25, 95%CrI (0.21, 1.19)], reflecting the same trend as the latency data. While the credible intervals crossed zero, we found more accurate responding for accented than non-accented words [95%CrI (−0.0401, 0.7006)] and less accurate response for the accented than for non-accented non-words [95%CrI (−0.7802, 0.0874)].

In sum, the effect of Format (accented vs. non-accented) was the opposite for words and non-words. The issue now is whether this dissociative pattern was due to a “word” bias for accented items in the yes/no lexical decision task. To examine this hypothesis, we designed an experiment parallel to Schwab (2015, Experiment 2). Participants only had to respond to words (i.e., go-no/go lexical decision) in a design composed of two blocks: one containing only accented items, and one containing only non-accented items (i.e., with an omitted accent mark for words, as in carcel). In this scenario, the presence of an accent mark would not bias participants to respond “word,” hence the difference between response times to words like cárcel and carcel is expected to be minimal.

## Experiment 2

### Materials and Methods

#### Participants

We recruited an additional sample of 40 participants from the same population as in Experiment 1.

#### Procedure

It was the same as in Experiment 1, except for the following: (1) participants had to press the “word” key if the item was a word and refrain from responding if the item was not a word; (2) the deadline for responding was reduced from 2 to 1.5 s to speed up no-go trials; and (3) the experiment was composed of two blocks: a block in which all the items (words and non-words) were accented and a block in which all the items were not accented (i.e., with an accent omitted for words, as in carcel). The order of the blocks was counterbalanced across participants. There was a brief practice phase (8 trials) before each of the blocks.

#### Materials

They were the same as in Experiment 1.

#### Results and Discussion

The statistical analyses were parallel to those in Experiment 1. The only difference was, due to the characteristics of the go/no-go procedure, we only obtained correct response times for word trials. Table 2 presents the mean correct response times and error rates per condition.

**TABLE 2:** Mean lexical decision times (in ms) and error rates (in percentages) for words and non-words with vs. without accent marks in Experiment 2.

	With accents		Without accents	
	Response time	% Errors	Response time	% Errors
**Words**	608	0.7	617	1.3
**Non-words**	–	3.5	–	3.1

In the latency data, we found a small 7-ms disadvantage for the words with the accent omitted relative to the words with the accent present. As the 95% credible interval of this difference crossed zero [*b* = 5.31, SE = 3.88, 95%CrI (−2.34, 12.86)], we prefer to interpret this pattern as a minimal/null effect.

In the accuracy data, we found higher accuracy for word trials than for non-word trials [*b* = −2.13, SE = 0.50, 95%CrI (−3.18, −1.20)]. More importantly, we did not find evidence of an effect of Format [*b* = −0.22, SE = 0.51, 95%CrI (−1.19, 0.82)] or an interaction between two factors [*b* = 0.25, SE = 0.50, 95%CrI (−0.78, 1.20)].

The present go/no-go lexical decision experiment, using exactly the same materials of Experiment 1, only revealed a negligible reading cost for the words with the omitted accent mark, thus replicating Schwab (2015, Experiment 2).

## General Discussion

We designed two lexical decision experiments to examine whether the omission of an accent mark in a Spanish word could have a genuine reading cost during lexical access. Recent research on this topic has failed to reveal a reading cost of omitting the accent mark across several procedures (masked priming lexical decision: Perea et al., 2020b; semantic categorization: Perea et al., 2021b; go/no-go lexical decision: Schwab, 2015). However, in a yes/no lexical decision task, Schwab (2015, Experiment 1) found a 29-ms disadvantage for the words with the omitted accent—the non-word data were not presented. Experiment 1 successfully replicated the advantage of cárcel over carcel (a 24-ms advantage) reported by Schwab (2015, Experiment 1). Critically, the analyses of the non-word data offered fundamental clues on the nature of this effect: response times to accented pseudowords were, on average, 26 ms slower than the response times to non-accented pseudowords (e.g., cardil < cárdil). That is, the pattern of non-word data was just the opposite of the word data, thus suggesting a “word” bias for accented items in the yes/no lexical decision task. Indeed, Experiment 2, using a go/no-go lexical decision task and a blocked design, showed similar response times to words regardless of whether the accent mark was present or omitted.

Thus, the present experiments have shown that the apparent processing disadvantage of words with an omitted accent mark in the yes/no lexical decision task in the present Experiment 1 (and Schwab’s Experiment 1) can be readily explained by the characteristics of the procedure. The dissociation between accented words vs. non-words fits very well with the second scenario given in the Introduction: items with an accent mark may be treated as more wordlike in a yes/no word/non-word decision. This mechanism would produce slower response times to words with the omitted accent (e.g., carcel > cárcel) and faster response times to pseudowords without an accent mark (e.g., cardil < cárdil). This type of dissociation in the yes/no lexical decision task is not new. A similar dissociation has been reported when manipulating other elements such as letter-case: in lexical decision, the word CAMINO (path) is responded faster than the mixed-case word cAmInO, whereas the pseudoword REVIDO is responded slower than the mixed-case pseudoword rEvIDo (e.g., see Perea et al., 2020a). Of note, the set of words employed in the present experiment only showed a minimal negligible reading cost when the accent mark was omitted in first-pass eye fixation measures during sentence reading (see Marcet and Perea, 2021) and in the response times in a task that requires on access to lexical-semantic memory (semantic categorization task, see Perea et al., 2021b). Therefore, the more parsimonious account of the present experiments is that the omission of accent marks in the yes/no lexical decision task does not hinder lexical access.

To sum up, our findings are consistent with the view that the omission of accent marks in Spanish—at least for words with unambiguous spelling—only conveys a negligible reading cost. This pattern has implications for the front-end of computational models of visual-word recognition in Spanish: non-accented vowels (e.g., a) can be represented at the level of abstract letter entries together with their accented counterparts (e.g., á). Nonetheless, we acknowledge that accent marks in Spanish may play some role at a phonological level—this may be more manifest in tasks that involve grapheme-to-phoneme associations (e.g., naming task), particularly for unfamiliar words. At a more applied level, the present experiment serves as another call to further simplify accentuation rules in Spanish—probably in the same line as in Italian. Finally, it is also essential to consider that the function of accent marks differs across languages, and the above conclusions may not apply to other languages (see Marcet et al., 2021, for discussion).

## Data Availability Statement

The datasets presented in this study can be found in online repositories. The names of the repository/repositories and accession number(s) can be found below: https://osf.io/w592x/?view_only=bb13458d70864b4ea10176be0de72bbc.

## Ethics Statement

The studies involving human participants were reviewed and approved by the Comité de Ética de Investigación en Humanos (CEIH) of the University of Valencia. The participants provided their written informed consent to participate in this study.

## Author Contributions

AM, MF-L, ML, and MP contributed to the conception and design of the study. ML and MP performed the statistical analyses. AM, MF-L, and MP wrote the first draft of the manuscript. ML wrote some sections. All authors contributed to the article and approved the submitted version.

## Conflict of Interest

The authors declare that the research was conducted in the absence of any commercial or financial relationships that could be construed as a potential conflict of interest.

## Publisher’s Note

All claims expressed in this article are solely those of the authors and do not necessarily represent those of their affiliated organizations, or those of the publisher, the editors and the reviewers. Any product that may be evaluated in this article, or claim that may be made by its manufacturer, is not guaranteed or endorsed by the publisher.

## Footnotes

1
www.pavlovia.org

2
www.limesurvey.org
